# Circulating biomarkers to monitor cancer progression and treatment

**DOI:** 10.1016/j.csbj.2016.05.004

**Published:** 2016-06-01

**Authors:** Suthee Rapisuwon, Eveline E. Vietsch, Anton Wellstein

**Affiliations:** Georgetown University Medical Center, Lombardi Comprehensive Cancer Center, 3970 Reservoir Rd, NW, Washington, DC 20007, USA

**Keywords:** cell-free DNA, cell-free RNA, cell-free microRNA, cell-free circulating nucleic acids, circulating tumor DNA, circulating mutant DNA

## Abstract

Tumor heterogeneity is a major challenge and the root cause of resistance to treatment. Still, the standard diagnostic approach relies on the analysis of a single tumor sample from a local or metastatic site that is obtained at a given time point. Due to intratumoral heterogeneity and selection of subpopulations in diverse lesions this will provide only a limited characterization of the makeup of the disease. On the other hand, recent developments of nucleic acid sequence analysis allows to use minimally invasive serial blood samples to assess the mutational status and altered gene expression patterns for real time monitoring in individual patients. Here, we focus on cell-free circulating tumor-specific mutant DNA and RNA (including mRNA and non-coding RNA), as well as current limitations and challenges associated with circulating nucleic acids biomarkers.

## 1 Introduction

**Fig. 1:**
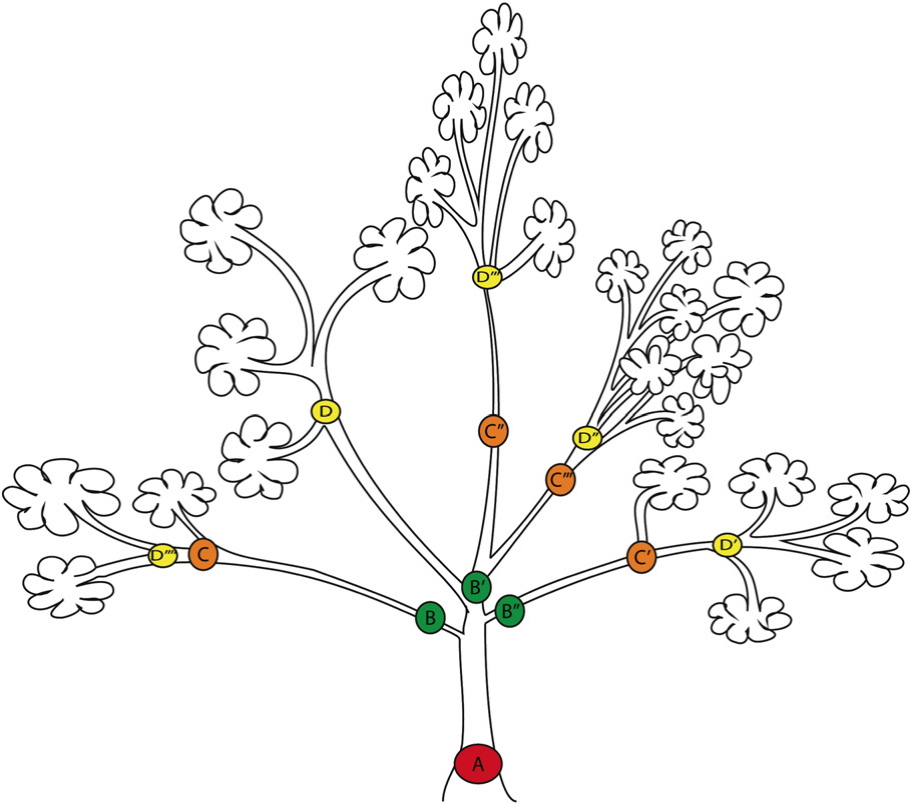
Branching of a cancer evolutionary tree. This model is similar to animals' phylogeny. A (red) represents a common tumorigenesis event, often characterizes by a common driver mutations. B (green) is the first, C (orange) and D (yellow) are subsequent branch evolutionary events.

**Fig. 2:**
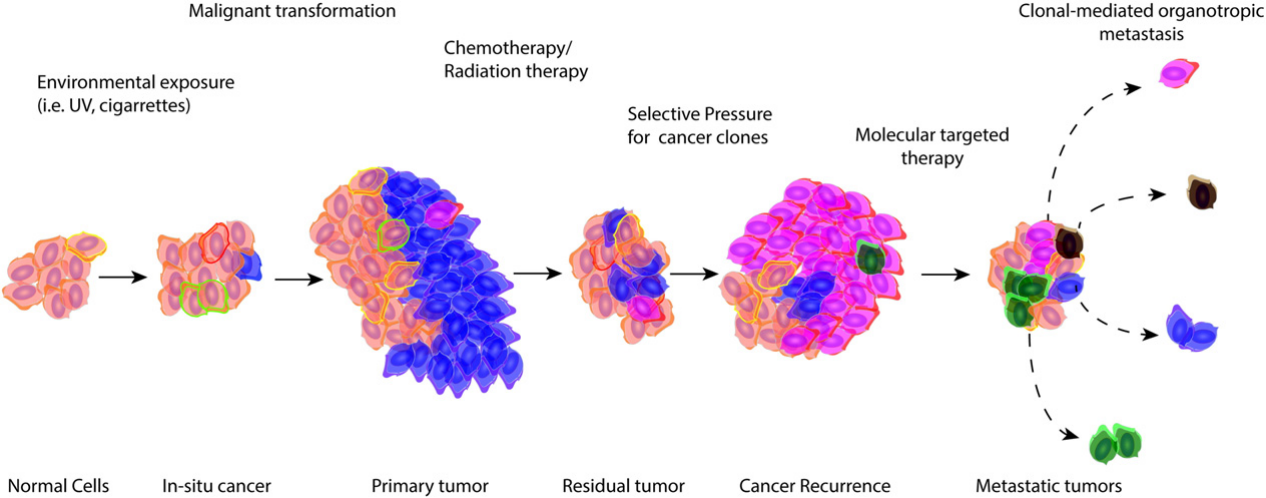
Selection of cancer subpopulation during tumor progression and treatment. Both genetics and environment factors influence tumorigenesis and cancer evolution. Selection will enhance cell growth, proliferation, invasion, metastasis, immune evasion and reduce apoptosis. Clones with unfavorable compositions of genetic or epigenetic alterations (blue) will be eliminated after primary therapy. Resistant clones (pink) with survival advantages are indicated. Orange: normal cells; colored-outline: pre-malignant lesion, blue, pink, green, dark brown: different malignant clones.

Tumor heterogeneity that enables malignant progression by evolutionary selection is also the major cause of emergent resistance during cancer treatment. Yet, we rely on few standard diagnostic tumor biopsies for the characterization of a given cancer. These specimens will provide only a partial characterization of the overall makeup of the dynamic systemic disease cancer represents with intratumoral and interlesional heterogeneity as well as emerging host responses [1]. Tumor heterogeneity is generally accepted as following Darwinian evolutionary principles (Fig. 1), where genetic heterogeneity within a cancer cell population translates into a range of phenotypes that includes distinct surface marker expression, metabolism, proliferation, apoptosis, invasion, angiogenesis, drug sensitivity, antigen presentation or organotropism of cell subpopulations present in a given tumor [2], [3]. Selective pressure and selection of cancer cell subpopulations are generally thought to drive increasing heterogeneity during tumor growth and metastatic spread (Fig. 2). Additionally, phenotypic plasticity of cancer stem cells in response to changes in the tumor microenvironment contribute to heterogeneity [4].

A striking example that illustrates intratumoral heterogeneity was recently described for kidney cancer specimen that revealed distinct expression of an autoinhibitory domain of the mTOR kinase and multiple tumor-suppressor genes (i.e. *SETD2*, *PTEN* and *KDMSC*). Additionally, this study demonstrated extensive heterogeneous mutational profiles in 26 out of 30 tumor samples from four renal cell carcinoma patients [5]. Another illustrative example of intratumoral/intermetastatic tumor heterogeneity is the extensive whole genome sequencing analysis of a patient with breast cancer and brain metastasis. Four different tissue samples (the primary tumor, blood, brain metastasis and xenografts) showed tumor heterogeneity at a low frequency even at the primary tumor [6]. Therefore, a single tumor biopsy will underestimate the mutational landscape due to intratumoral/interlesional mutational and phenotypic | heterogeneity. These concepts and additional examples were reviewed recently [7].

## 2 What are circulating biomarkers

Capturing and analysis of circulating biomarkers is an alternative method to gain insight into the molecular makeup of a cancer in a given patient. Historically, circulating biomarkers have been observed and studied since the late 1800s in a form of circulating tumor cells (CTCs) [8]. However, extensive study on CTC did not occur until the mid-20th century when the studies of circulating tumor cells showed that the presence of CTCs in cancer patients was correlated with poorer prognosis or progression-free and overall survival [9], [10], [11].

**Fig. 3:**
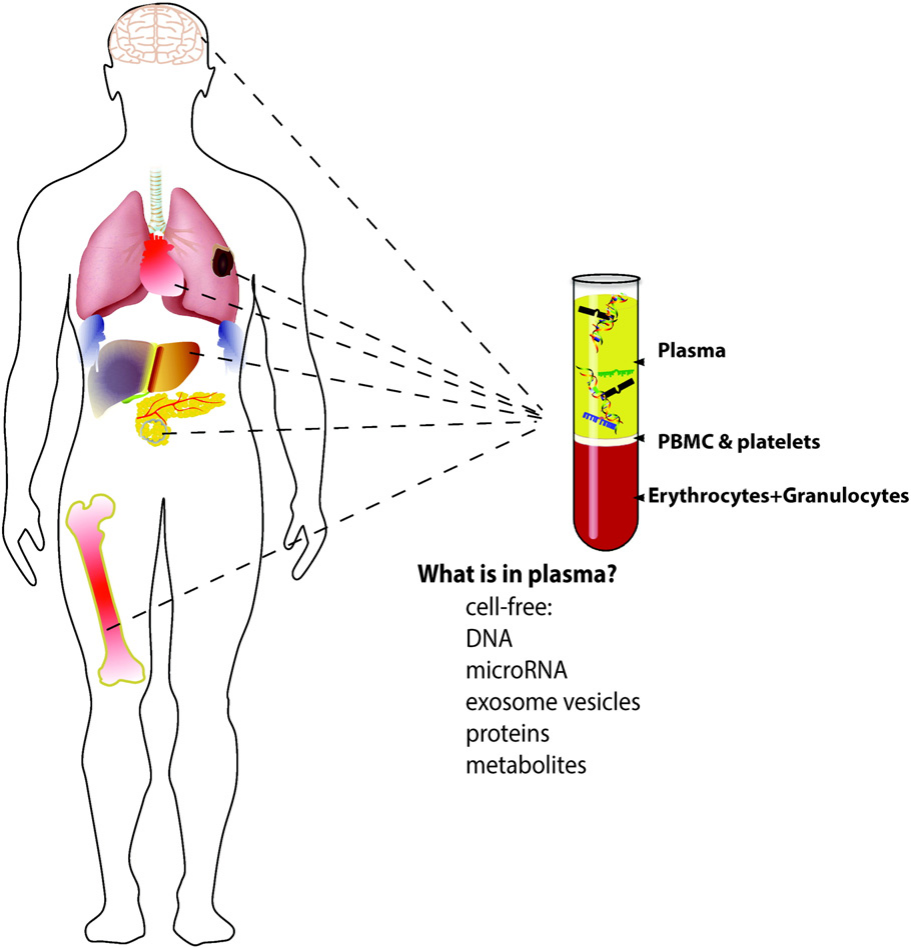
Circulating biomarkers. Circulating cell-free (plasma/serum) biomarkers include nucleic acids, extracellular vesicles, proteins and metabolites from all metastatic sites as well as normal organ physiologic turn over or impact of systemic drug treatment. Each organ contributes wild-type DNAs to the circulation and organ metastatic seeds will shed mutant DNA. Circulating microRNAs, exosomal RNAs and long non-coding RNAs thus reflect the overall host-tumor crosstalk.

Here we will discuss cell-free circulating tumor-specific mutant DNA and RNA (including mRNA and non-coding RNA; Fig. 3) due to recent improvements in the sensitivity and analysis scope that impacted the potential of these approaches significantly. A review of circulating tumor cells, circulating proteins, and metabolites will not be included here.

## 3 Circulating tumor DNA (ctDNA)

Circulating, cell-free DNA (cfDNA), i.e. fragments of DNA found in the cell-free blood compartment was first described in 1948 [12], but cell-free DNA fragments that originated from tumor cells (ctDNA) have not been well characterized until the late 1980s [13]. The origin of ctDNA has not been well defined yet, but is thought to result from cell death. The presence of ctDNA has been correlated with overall tumor burden, and disease activities [14], [15]. Somatic oncogenic Ras, p53 and other cancer-related gene mutation, promoter hypermethylation of tumor suppressor genes have been detected and measured in several different cancers including, but not limited to, colon, small cell and non-small cell lung cancer, melanoma, kidney and hepatocellular carcinoma [16].

It is believed that ctDNAs are results of apoptosis. Nucleosomes play essential roles in the fragmentation of DNA during programmed cell death and a recent study developed a genome-wide nucleosome map that showed ctDNA fragments bearing footprint of transcription factors in specific tissues [17]. Additionally, ctDNA from cancer patients also demonstrated distinct pattern of nucleosome spacing which suggested contribution of ctDNAs from non-hematopoietic tissues, unlike ctDNAs from healthy counterparts whose contribution of nucleosome spacing are mostly from lymphoid and myeloid tissues.

## 4 Detection methods and sensitivity

ctDNA detection methods have improved substantially during the past few decades. In the early 1990s, recovery of ctDNA was performed by conventional polymerase chain reaction, followed by Sanger sequencing. However, recovery of ctDNA was often inconsistent, and was considered inferior to other biomarkers, including circulating tumor cells (CTCs) and cancer-related protein markers (i.e. alfa-fetoprotein, lactate dehydrogenase). The main obstacle in the detection of ctDNA is the relatively low abundance per milliliter of blood examined. Conventional methods of PCR detection and Pyrosequencing have their lower limit of detection at 10% of ctDNA copies in the bulk of background normal DNA (Table 1). Similarly, the early 2000s method of Next-generation sequencing and quantitative PCR (qPCR) lowered the lower limit of detection to approximately 1–2% and enhanced detection performance in hematologic malignancies i.e. Bcr-Abl fusion transcripts in chronic myelogenous leukemia from circulating leukemic cells. Nevertheless, the detection of ctDNA in patients with solid tumors using these techniques remained problematic. The first and successful molecular technique in the identification of ctDNA was the introduction of Beads, Emulsion, Amplification and Magnetics (BEAMing) [18], [19] that consisted of emulsion PCR and included Streptavidin-coated beads in every PCR compartment, followed by recovery of tagged amplicons and fluorescent oligohybridization of the mutation of interest. (See Table 2.)

More recent methods using droplet digital PCR [20] and targeted panels of amplicon sequencing [21] platforms improve ctDNA recovery and further decrease the lower limit of detection to approximately 1 in 10,000 copies (0.01%). Droplet Digital PCR (ddPCR) takes advantage of partitioning the PCR amplification reactions into approximately 10,000 to 20,000 independent polymerase reactions per tube. This bypasses both reverse transcription, amplification efficiency, and avoids the need for data normalization between each sample [22] according to the Minimum Information for Publication of Quantitative Real-Time PCR Experiments (MIQE) guidelines, both of which are prone to analytical error. Direct measurement of mutant DNA copies further minimizes errors in relative quantification of qPCR and streamlines the analysis with less additional steps.

PCR-based assays do carry limitations related to their detection methods. The numbers of ctDNA that can be detected in one assay are limited. The number of fluorescence acquisition channels available often dictates the number of multiplex-droplet PCR amplification and probe-hybridization reactions. BEAMing is labor-intensive and requires both Streptavidin bead emulsion PCR and flow cytometry, thus, decreasing productivity and the possibility for high-throughput analyses. Also, only known targeted mutations are measured in BEAMing or ddPCR analysis. This also generates a challenge in situations where the amount of template DNA is limited and multiple mutations may be emerging.

Genome wide approaches to assess global ctDNA in the circulation have gained significant attention. This is because only a fraction of patients has known cancer-related driver mutations, i.e. *EGFR*, *BRAF* or *KRAS*. However, initial efforts to utilize shot-gun approaches with whole-exome sequencing to identify and measure ctDNA were difficult due to ctDNAs being fragmented and degraded in the circulations. This further complicates the validation of variant calling in extensively fragmented DNA samples [23]. A new method that utilized multiple-tiered mutation analysis based on somatic mutation found in non-small cell lung cancer in The Cancer Genome Atlas (TCGA), i.e. cancer personalized profiling by deep sequencing (CAPP-Seq) [24], have improved ctDNA detection. In a set of 96 patients with stage II–IV NSCLC the authors reported 96% specificity for mutant allele frequency with lower limit of detection at 0.02%. This method remains dependent on tumor volume and the type of cancer assessed due to differences in quantifiable ctDNA that is distinct between cancer types.

## 5 Clinical application

ctDNA are found at a relatively high concentration in the peripheral circulation in patients with metastatic cancer, compared with localized disease [16]. Also, the presence and amount of ctDNAs in the circulation is independent of the presence or concentration of CTCs [16], suggesting independent mechanisms of shedding ctDNA and CTCs. Moreover, the ctDNA concentration reflects the response to chemotherapy, or molecular targeted therapy [25], [26]. These findings will still need to be tested for their clinical implications.

## 6 Cancer screening

Conventionally, cancer-related protein markers have been used to monitor patients with limited sets of cancers for recurrent disease, i.e. CA-125 in ovarian cancer, AFP for hepatocellular carcinoma, carcinoembryonic antigen (CEA) for colorectal adenocarcinoma, or lactate dehydrogenase (LDH) for malignant melanoma. Unlike germ-cell tumors where cancer-related protein markers are highly sensitive and specific to cancer activities, the majority of cancer-related proteins, i.e. LDH, remain only screening tools for cancer recurrence without adequate specificity.

ctDNA are more abundant in the circulation among metastatic cancers than early-staged disease, and the prevalence of ctDNA detected in patients with no radiographic evidence of metastasis varies between 49–78%, compared with 86–100% in metastatic disease [27].

Alternative method to monitor disease activity is through the detection of unique sets of single nucleotide point mutations specific to the patient as indicators of disease activity. Also, identification of a patient's specific somatic chromosomal translocation through high-throughput sequencing, (“personalized analysis of rearranged ends” PARE) or through next-gen, matched-pair sequencing analysis have recently been established. [28], [29], [30], [31] This approach uses tumor-specific somatic rearrangement as personalized biomarkers to monitor disease activities with the notion that all tumor cells carry structural chromosomal rearrangements that are not presented in normal tissue or in the circulations. Major potential limitations in this personalized biomarker monitoring includes the stability of each biomarker during the treatment course as the detected biomarker could possibly represent passenger mutations/rearrangements that can undergo negative selection and disappear as the tumor progresses.

## 7 Prognostic markers

Earlier studies used restriction fragment-length polymorphism and polymerase chain reaction (RFLP-PCR) assays on circulating DNA to selectively detect circulating mutant *KRAS* in patients with non-small cell lung cancers. This correlated with the presence of KRAS mutations in tumors and with poorer prognosis for overall survival [32]. Several subsequent studies have confirmed the positive correlation between survival and ctDNA burden using newer and more sensitive detection methods. For example, in a cohort of 69 patients with metastatic colorectal cancers with detectable *KRAS* ctDNA, the higher concentration of ctDNA correlated with a poorer survival rate, independent of ECOG performance status, and CEA level [27]. Another series also demonstrated the prognostic significance of increased levels of ctDNA that is related to poor overall survival in patients with metastatic breast cancer, a relationship that cannot be found between level of CA15-3 and metastatic breast cancer survival [28], [33]. Relationship of the ctDNA concentration has been linked to disease burden, prognosis, and response to therapy. The utility of ctDNA as a prognostic biomarker has been extended to different type of cancers, for example cervical cancer [34], colorectal cancer [35], [36], pancreatic cancer [37], [38], [39], and melanoma [40], [41].

## 8 Predictive markers

Predictive biomarkers that can guide treatment decision have been sought after to identify subsets of patients who would be “exceptional responders” to specific cancer therapies, or individuals who would benefit from alternative treatment modalities. An example of ctDNA as a potential predictive biomarker is the measurement of O^6^-methyl-guanine-methyl-transferase (*MGMT*) promoter methylation from ctDNA in glioblastoma multiforme (GBM) patients. This would determine potential benefits from adjuvant alkylating chemotherapy such as temozolomide or dacarbazine, in addition to standard post-operative adjuvant radiation [42], [43]. Identification of plasma ctDNA with *MGMT* methylation using methyl-BEAMing and bisulfite-pyrosequencing techniques in metastatic colorectal cancers demonstrated 86% agreement of *MGMT* methylation status the tumor and ctDNA analyses with the most methylated allele in the tissues presented in the circulation. Additionally, *MGMT* methylation status in ctDNA was associated with improved median PFS (2.1 v.s. 1.8 months; p value: 0.08) [44]. Analysis of tumor specific ctDNA could thus facilitate the detection of emerging resistant mutations to molecular targeted therapy, and could help tailor the appropriate treatment based on mutations detected in the tumor or in the circulation. Sundaresan et al. [45] demonstrated that the use of ctDNA, complemented by mutation analyses of CTCs and tumor biopsies can improve the detection rate of T790M EGFR resistant mutation to molecular targeted therapy of non-small cell lung cancers, first- and second-generation EGFR tyrosine kinase inhibitors.

ctDNA can also be incorporated into prospective clinical studies to identify predictive markers of response to cancer therapy with stratifications based on the underlying somatic mutation that will render subjects susceptible to specific targeted therapies. (e.g. BRAF L597 mutation in cutaneous melanoma with MEK inhibitor, or PIK3CA mutation in solid tumors with PIK3CA inhibitors) or indicate emerging resistant subclones.

## 9 Treatment monitoring

Several studies have utilized ctDNAs as markers of metastatic disease activities to monitor disease response and overall disease burden. In one study, a total of 30 out of 52 patients with metastatic breast cancers were found to have somatic variants in their tumors, either by targeted gene sequencing, or whole-genome paired-end sequencing. Compared with CTCs and CA 15–3, 97% of patients had measurable ctDNA, compared with 78% for CA 15–3, and 87% for CTCs. The trend of serial ctDNA levels appeared to correlate with radiographic response to therapy. A comparison showed fluctuations of CTCs that are not informative when the number of CTCs was below 5 cells/ml, and CA 15–3 changes in response to cancer treatment were only small.

Application of ctDNA for treatment monitoring and surveillance could be useful in certain malignancies where there is no optimal method of screening and surveillance, such as pancreatic cancer, or ovarian cancers. Pereira et.al [46] suggested the potential utility of ctDNA as an early screening and surveillance tool for gynecologic malignancies (22 ovarian cancers, 17 endometrial cancers, three fallopian tube cancers, one peritoneal cancer, and one synchronous fallopian tube and uterine cancer), where CA-125, an existing protein biomarker, is neither sensitive nor specific to inform treatment decision. In this study, patient-specific mutations discovered from exome and targeted amplicon sequencing of each tumor were then recovered in the peripheral circulation as ctDNA at a 93.8% detection rate. Furthermore, the presence of ctDNA provided an average lead-time of seven months over computed tomography (CT) scans.

## 10 Limitations

While ctDNA monitoring could offer potential improvements in non-invasive cancer treatment monitoring, there are inherent limitations related to ctDNA tumor markers. ctDNAs demonstrates a strong correlation with tumor burden but are not always detectable in peripheral blood. Most studies have shown an approximately 70–80% concordance between tumor somatic mutation and the presence of ctDNA in the circulation [25], [47].

ctDNA quantification is highly dependent on pre-analytical specimen handling. While it is possible to recover ctDNA at a comparable concentration between 2–4 h and 24 h processing time [25], [48], several studies have demonstrated significant changes in the mutant-to-wild type DNA ratios between specimens processing within 2–4 h of blood collection relative to processing at 24 h. There is also no consensus on the method of ctDNA quantification and how ctDNA should be selected from multiple mutations detected in the cancer genome.

The source of ctDNA should also be standardized, either from serum or plasma. Prior studies [49], [50] demonstrated a discrepancy of ctDNA concentrations between serum and plasma samples. ctDNA concentrations were consistently low in the plasma compared to the serum due to possible loss of circulating DNA during purification, as coagulation and other proteins are being eliminated during specimen preparation.

While ctDNA could be useful in the early detection of cancer recurrence, a potential major limitation is the lack of a consensus on the next step of management following detection of ctDNA in individuals without radiographic evidence of cancer recurrence or relapse. A great example has been CA-125, a protein biomarker for ovarian cancer, in the MRCOV05/EORTC 55955 trial [51] for which 529 of 1442 ovarian cancer patients completed their chemotherapy and had their CA-125 returned to normal range were randomized to either early or delayed treatment upon their recurrence of CA-125 above twice normal limits. Despite earlier treatment based on elevated CA-125 level, there was no difference in overall survival (median overall survival 25.7 months (95% confidence interval (CI), 23.0–27.9) in the early treatment arm vs. 27.1 months (95% CI, 22.8–30.9) in the delayed treatment arm, with a hazard ratio (HR) of 0.98 (95% CI, 0.80–1.20; p = 0.85). This finding led to a recommendation against treatment decision based on CA-125 alone without radiographic or physical evidence of disease recurrence.

Similarly, lead-time bias is another major challenge in early cancer screening tools, as previously mentioned in yearly low-dose CT scan for lung cancer screening, and routine PSA monitoring in prostate cancer [52], [53], [54]. Further research should be performed to validate the utility of ctDNA as potential biological markers in prospective trials.

## 11 Circulating RNA

### 11.1 Types of circulating cell-free RNA: messenger RNA

Circulating messenger RNAs (mRNA) in human cancer patients were first described in the 1990s in patients with different type of cancers, i.e. gastric cancer, pancreatic cancer [55], nasopharyngeal carcinoma [56] and melanoma [57]. Because mRNAs possess a critical role in intracellular protein translation and, it is likely that extracellular mRNAs reflect the status of the intracellular process, and are conceivably potential biomarkers for cancer diagnosis or therapeutic monitoring. Later studies reported various coding RNAs in plasma or serum from patients with cancer, and levels of circulating cell free mRNAs (cf-mRNA) were found to be predictive of clinical outcome [58], [59] and disease prognosis [60], [61]. However, extracellular circulating mRNAs are subjected to degradation, instability, low abundance, and intracellular mRNA contamination from specimen processing [62], [63]. Thus, the reproducibility and utility of cf-mRNA as biomarkers is severely limited.

### 11.2 Types of circulating cell-free RNA: non-coding RNA

Non-coding DNA sequences are actively transcribed into non-coding RNAs consisting of long non-coding RNAs (lncRNA), microRNAs (miRNA), short interfering RNAs (siRNAs), and piwi-interacting RNAs (piRNA), among other lncRNA species. Unlike mRNA, the function of non-coding RNA is the regulation of gene expression. The vast majority of observations in the field of circulating RNAs involve miRNAs and lncRNAs, however as increasing RNA sequencing data is being generated, it is becoming clear that piRNAs and snoRNAs in human plasma are gaining importance.

### 11.3 Piwi-interacting RNAs (piRNA)

PiRNAs are single stranded 26–31 nucleotide long RNAs which can repress transposons and target mRNAs, mediated by binding to PIWI proteins. PIWI proteins belong to a subfamiliy of Argaunate proteins. piRNA biogenesis is Dicer and Drosha independent [64] Although the piRNAs are studied only recently, it is known that piRNAs are a large class of small non-coding RNAs in animal cells and it is thought there are many thousands of distinct piRNAs. According to the piRNABank (http://pirnabank.ibab.ac.in/stats.html) there are more than 32,000 unique piRNAs. In addition to their role in maintaining the integrity of germ line DNA, piRNAs are found to be deregulated in cancer. [65] PiRNAs are highly abundant in human plasma [63] Plasma levels of PiR-019825 were found to be deregulated in patients with colorectal cancer, whereas piR-016658 and piR-020496 were associated with prostate cancer patients, and plasma levels of piR-001311 and piR-016658 were found to be dysregulated in patients with pancreatic cancer. [63] Despite their large quantities, the role of piRNAs in the circulation has not been studied and still needs to be elucidated.

### 11.4 Small nuclear and small nucleolar RNA(snRNA and snoRNA)

snRNA and snoRNA consist of large number of non-coding RNA species of 60–300nucleotide long that were transcribed from intervening sequences of protein-coding genes (a.k.a.host genes). snRNAs are important in RNA-RNA remodeling and spliceosomes assembly. snoRNAs involve in post-transcriptional modification of ribosomal RNA and play integral roles in formation of small nucleolar ribonucleoprotein particles (snoRNP), which are important cellular regulation and homeostasis. There are two major classes of snoRNA, the first one is box C/D snoRNA, a.k.a. SNORDs (contains box C (RUGAUGA) and D (CUGA) motif), and box H/ACA snoRNA, a.k.a. SNORAs, (contains box H (ANANNA) motif and ACA elements) (review in [66]). Perturbation of snRNA and snoRNA expression has been documented in different type of cancers. Increased ratio of U6 snRNA to SNORD44 snoRNA were noted to be higher in breast cancer patients regardless of disease status or staging. SNORD112–114 are overexpressed in acute promyelocytic leukemia and suppression of the same snoRNAs under the effect of all-trans retinoic acid-mediated differentiation [67]. There are also enrichment of U22, U3, U8, U94 box C/D snoRNAs in human breast cancer cell lines [68] and over-expression of both SNORD and SNORA species in lung adenocarcinoma and squamous cell carcinoma [69], [70]. Additionally the same study demonstrates increased expression of certain snoRNAs species. Namely, in a study of snoRNA on non-small cell lung carcinoma that showed increased expression of SNORD33, SNORD66, SNORD73B, SNORD76, SNORD78, and SNORA41, subsets of overexpressed snoRNA, SNORD33, SNORD66, SNORD76 were reliably detectable in the NSCLC patients' plasma at a significantly higher level compared to healthy controls or COPD patients. However, there remains paucity of data on snRNAs and snoRNAs as potential diagnostic, prognostic or predictive markers.

### 11.5 Long non-coding RNAs (lncRNA)

The lncRNAs are defined as > 200 nucleotides in length and classified into five subclasses, which include intergenic, intronic, sense overlapping, *anti*-sense, and bidirectional lncRNAs [71]. LncRNA regulates expression of protein-coding genes, functions at the level of splicing, chromatin remodeling, transcriptional control and post-transcriptional processing after binding to DNA, RNA or proteins [72]. Dysfunction of lncRNAs is associated with a wide range of diseases. Experimentally supported lncRNA-disease associations are collected and curated in publicly available domain, i.e. the LncRNADisease database which contains sequence annotations, description of lncRNA functions and organ specific expression levels [73]. LncRNADisease also curated lncRNA-interacting partners at various molecular levels, including protein, RNA, microRNA and DNA. Several thousand RNA transcripts have been identified as lncRNAs [74] and their expression are tissue-specific [75], involving growth, metabolism and cancer metastasis [76]. Despite the paucity of data on circulating lncRNAs, the interest in circulating lncRNAs in human cancer has grown recently [77], [78], [79], [80], [81]. In renal cell cancer, levels of plasma lncARSR is higher than those of healthy blood donors, lncARSR levels decreased after tumor resection and were elevated upon tumor relapse. [82]. Moreover they showed that high pre-therapy plasma lncARSR levels could predict which patients would suffer from progressive disease during sunitinib therapy. This could indicate that circulating lncRNAs have potential to serve as predictive biomarkers for clinical benefits of cancer therapy.

Interestingly, the ratio of different RNA transcripts within exosomes differs from their cells of origin, suggesting that lncRNA are transported into exosomal vesicles in a tightly regulated manner [83]. For example, circulating levels of lncRNA H19 are elevated in patients with gastric cancer compared with healthy controls and plasma H19 lncRNA expression was reduced postoperatively in patients with elevated levels of H19 lncRNA pre-operatively [84]. However, there was no correlation between the expression of H19 in plasma and primary tumor tissues. This discrepancy may be due to decreased RNA integrity in plasma and reduced RNA quality and degradation in formalin-fixed paraffin-embedded (FFPE) tissues. Interestingly, there was no difference in H19 expression between tumor and paired non-cancerous tissues in FFPE samples. These findings provide evidence of different tissues of origin from each circulating lncRNAs, e.g. the lymphatics, the cardiovascular or nervous system, circulating peripheral blood cells or hematologic stem cells. This implies that circulating lncRNAs can provide information about the tumor-host microenvironment and crosstalk, and thus reflect the systemic nature of cancer. A study using sera from gastric cancer patients suggested that circulating CUDR, PTENP1 and LSINCT-5 lncRNAs expression could distinguish patients with gastric cancer as early as stage 1 from healthy subjects and from patients with gastric ulcers, although there was no association between the lncRNAs and tumor characteristics (location, size, and TNM staging) [85].

### 11.6 microRNA

Mature microRNAs (miRNA) are highly conserved short strands of non-coding RNA, derived from hairpin precursor transcripts [86]. After cleavage of primary microRNA (pri-miRNA) transcripts by the Drosha/DCGR8 complex, nuclear-to-cytoplasmic transport, and maturation with DICER1 [87], [88], 21–24 nucleotide long, double stranded mature miRNAs are formed. One of the mature miRNA strands binds predominantly to the 3′untranslated region (UTR) region of mRNA to regulate protein translation. Additionally, miRNAs can also bind to the open reading frame (ORF) or 5′UTR of target mRNAs to repress or activate translational efficiency [89], [90], [91], [92]. The discovery of small RNAs that are involved in translation regulation via an antisense RNA-RNA interaction was first described in *Caenorhabditiselegans*[93]. To date, more than 2500 human mature miRNAs have been identified and annotated [94], with more than half of human protein-coding genes likely regulated by a miRNA [95].

miRNAs are dysregulated in cancer and play crucial roles in cell proliferation, apoptosis, metastasis, angiogenesis and tumor-stroma interactions [96]. Dysregulated miRNA(s) can function both as oncogenes (e.g. miR-155; miR-21, miR-221; miR-222, miR-106b-93-25 cluster; the miR-17-92 cluster) and tumor suppressors (e.g. miR-15; miR-16; let-7; miR-34; miR-29; miR-122, miR-125a-5p and miR-1343-3p), depending on their downstream targets [63], [97]. Many human miRNA genes are located on chromosomal sites that are susceptible to chromosome breakage, amplification and fusion with other chromosomes [98]. Additionally, alterations in RNA binding proteins and cell signaling pathways contribute to cancer through miRNA expression changes as well as mutations in core components of the miRNA biogenesis machinery that can promote oncogenesis [87]. It has recently been shown that mutant *KRAS* in colon cancer cell lines leads to decreased Ago2 secretion in exosomes and Ago2 knockdown resulted in decreased secretion of let-7a and miR-100 in exosomes whilst cellular levels of the respective miRs remained unchanged compared to control cells. [99].

A systematic expression analysis of 217 mammalian miRNAs from 334 samples, including multiple human cancers revealed extensive diversity in miRNA expression across cancers, and a large amount of diagnostic information encoded in a relatively small number of miRNA. More than half of the miRNA (129 out of 217) had lower expression levels in tumors compared to normal tissues, irrespective of cell types [100]. miRNA expression profiles allows classification of poorly differentiated cancers and identify tumors of unknown tissue origin [100]. In subsequent studies, profiling miRNA expression improved cancer diagnosis and helped identify the tissue of origin in carcinoma with unknown primary site by standard histology or immunohistological analyses [101], [102].

miRNAs are present and stable in the peripheral circulation. The first report on miRNA expression in the circulation in 2008 described detection of four placenta-associated miRNAs (miR-141, miR-149, miR-299-5p, and miR-135b) in maternal plasma during pregnancy, after which the level decreased following delivery [103]. In 2008, a study demonstrated increased levels of circulating miR-21, miR-155 and miR-210 expression in patients with diffuse large B-cell lymphoma (DLBCL) compared to healthy controls [104]. Mitchell et al. also showed that circulating serum miR-141 could distinguish patients with advanced prostate cancer from healthy controls [105].

The vast majority of research on circulating miRNA signatures in oncology is focused on diagnostics [106], in which patients with cancer are compared to healthy individuals. Given the profuse inter-individual differences in genetic background of individual patients in addition to the heterogeneous nature of cancer, using cf-miRNA as cancer diagnostic biomarkers will remain challenging.

The origin of cf-miRNA is heterogeneous. miR-21 is a good example to illustrate this point. Although the release of miR-21 into the circulation is correlated with a multitude of cancer types, it is also highly expressed in activated T-cells and associated with inflammation and wound healing [107], [108], [109]. Elevated circulating miR-21 levels do not merely reflect tumor presence. They can also reflect the host response to the tumor, which is important in predicting disease progression. Moreover, there are often discordances between cf-miRNA signatures and the paired tumor tissue [106]. Assuming that the quality of miRNA measurements is not determined by the efficacy of RNA extraction, this suggests that cancer-associated cf-miRNA deregulations is more likely to reflect the systemic response to the presence of cancer. Indeed, several studies have shown that cf-miRNAs are predominantly derived from blood cells [110] and the endothelium [111] in addition to the tumor.

Cancer progression and systemic drug therapy involve many organ systems and are not limited to the primary tumor. This makes cf-miRNA attractive biomarkers for cancer progression and drug efficacy monitoring. For instance, in serum obtained pre-surgically from patients with early stage colorectal cancers, a panel of 6 circulating miRNAs can predict cancer recurrence [112]. Changes in cf-miRNA patterns within the same patients can be monitored over time during therapy. The growing evidence of the utility of cf-miRNA as cancer therapy response indicators has been accumulating during the last few years [113], [114], [115]. Cf-miRNAs are likely to surpass the clinical utility of conventional protein markers such as CA-125, CA19-9, PSA and radiographical techniques, which have low sensitivity and specificity and are not designed to characterize cancer at a genetic level.

## 12 Modes of RNA transport into the circulation

Human serum contains ribonucleases (RNase) that originate from leukocytes and the pancreas and catalyze the cleavage of bonds between ribonucleotides. Levels of serum RNases are elevated in patients with cancer [116]. Despite the rich abundance of RNAses, circulating RNAs have been found to be unexpectedly stable against RNase degradation, as long as the uncentrifuged blood is stored at 4 °C, and plasma is processed within 6 h. Also, single freeze/thaw cycle produces no significant effect on the RNA concentration of plasma or serum [117].

One explanation for the circulating RNAs' stability is encapsulation by protective membrane bound vesicles. These vesicles consist of a lipid bilayer membrane surrounding a small cytosol and are separated into three types: exosomes, microvesicles (MVs, ectosomes or microparticles), and apoptotic bodies (ABs). Each vesicle type can originate from normal or cancerous cells, transfer molecular cargo to both neighboring and distant cells, and modulate cellular behaviors involved in physiology and pathology [118], [119], [120].

Exosomes were first identified as vesicles with 5′nucleotidase activity in 1981 by Trams et al. [121] and later described as 30 to 100 nm vesicles of endosomal origin [122]. An attempt to profile the ribonucleic material enclosed within exosomes isolated from plasma of 3 healthy human blood donors was performed by using small RNA sequencing libraries designed to capture small non-coding RNAs of ~ 20–40 nucleotides length [123]. This analysis was recently repeated in a larger cohort of human subjects and generated similar results: The plasma exosomal RNA species are made up of 40.4% mature miRNAs, 40% piRNAs, 2.1% mRNAs and 2.4% lncRNAs [63]. In a recent RNA sequencing analysis in human plasma from 40 individuals, 669 miRNAs, 144 piRNAs and 72 snoRNAs were found to be expressed above one read per million [124].

Interestingly, bovine miRNAs were detected in the human plasma exosomes. However their origin remains to be elucidated since it is unknown whether dietary miRNAs can enter the human circulation through the gastrointestinal system. Microvesicles are larger vesicles (50 to 1000 nm) created through direct budding from the plasma membrane and contain metalloproteases in addition to lipids, cytokines, growth factors, membrane receptors and nucleic acids which exosomes also carry [119]. Exosomes can be separated from vesicles of different sizes using ultracentrifugation at different speeds, with the larger vesicles pelleting at lower speed than the smaller ones [118]. ABs are 500 to 2000 nm in diameter that are released by cells undergoing apoptosis and may contain genomic DNA fragments and histones in addition to RNAs. Tumor-derived mRNA associated with apoptotic bodies remains stable in serum, in contrast to mRNA in serum samples mixed with free tumor cell-derived mRNA even when the mRNA was rapidly extracted, i.e. within 1 min after incubation [125], [126]. Extracellular vesicles play a critical role in cancer, since they can contain oncogenes, mutated tumor suppressor genes, hypoxia-related molecules, angiogenic factors, immune regulatory proteins, RNAs, and various metabolites and the field of extracellular vesicle research in cancer biology is expanding fast.

Despite the protection provided by extracellular vesicles against RNA degradation, miRNA in plasma can pass through 0.22 μm filters and remain in the supernatant after ultracentrifugation, indicating the non-vesicular origin of a portion of extracellular miRNA [127]. This phenomenon is explained by the fact that miRNA can be transported when bound to proteins, in addition to being carried by vesicles. One example of miRNA delivering proteins is high-density lipoprotein (HDL). HDL can carry both exogenous and endogenous miRNAs to recipient cells resulting in direct targeting of mRNA reporters [128], and HDL-mediated delivery of miRs to recipient cells is dependent on scavenger receptor class B type 1. Furthermore, Nucleophosmin (NPM1, nucleolar phosphoprotein b23, numatrin) is thought to be involved both in the miRNA exporting process and in protecting external miRNAs outside the cell from RNAse digestion [129]. Another study describes that a large portion of plasma miRNAs cofractionated with protein complexes rather than with vesicles and that miRNAs were sensitive to protease treatment of plasma, indicating that protein complexes protect circulating miRNAs from plasma RNases [130]. Argonaute2 (Ago2) is present in plasma and is the key effector protein of miRNA-mediated RNA silencing. Importantly, the identification of extracellular Ago2-miRNA complexes in plasma raises the possibility that cells release a functional miRNA-induced silencing complex into the circulation. Irrespective of the packaging of circulating RNAs, extracellular RNA secretion is an active and tissue-specific phenomenon, which makes them biologically significant. It is likely that isolation of RNA from plasma or serum without prior separation into subsets can capture all compartments including membrane-derived vesicles and protein bound molecules. Since they are biologically functional regardless of the type of carrier, analysis of the complete assemblage should be performed for their utilization as informative biomarkers.

## 13 Method of detection for circulating RNA

There are multitudes of commercial RNA isolation kits available that serve their purpose adequately [131]). Cf-RNA yields are low compared to levels of RNAs of cellular or tissue origin, and depending on the desired type of RNA, diverse methods can be used for either total RNA or exclusively small RNA isolation.

The gold standard for RNA quantitation is qRT-PCR and this applies to circulating RNAs as well. The required input for this assay is as low as a few nanograms of RNA which makes qRT-PCR attractive for low abundant cf-RNA detection. Whether based on Taqman, Locked-Nucleic-Acid or Sybr-Green technology, overall RNA-specific qPCR is sensitive, the specificity of the assay is high and results are obtained within a day. A relatively novel technology called Droplet Digital PCR (ddPCR, Bio-Rad™) is described above and can also be applied to RNA. This analysis enables highly reproducible quantitation of low abundant RNAs. The limitations of RNA-specific qPCR are the low throughput, lack of suitable housekeeping gene normalizers and the inability of miRNA discovery.

Broad gene expression arrays allow for higher throughput, as they can include several hundreds of target RNAs in a single assay. Arrays are either based on qPCR or hybridization technologies and are commercially offered by ABI, Agilent, Affymetrix, Exicon, Nanostring, Toray, MiRXES and Illumina among many other companies. These assays require 30–100 ng RNA input. It has been reported that qRT-PCR-based arrays performed better than hybridization platforms with respect to limits of miRNA detection [132]. Adequate data normalization and analysis requires experience and can take several days.

For RNA discovery beyond detection of known target genes, RNA sequencing is necessary. However, cDNA library preparation may introduce sample bias. Deep sequencing with the use of small RNA-cDNA libraries is suitable for shorter RNAs, however for adequate mRNA and lncRNA transcript discovery, longer read sequencing may be more suitable.

Recently, next-generation sequencing (NGS) has emerged as an unbiased alternative option with greater dynamic range of detection, increased sensitivity and reproducibility. NGS platform could overcome fundamental problems with array-based platform that rely on hybridization of RNAs to the pre-specified probes and rendered small dynamic range of detection as well as limitation in discovery of new ncRNA species. It is also important to realize that cross-comparison of ncRNA, especially miRNA, between different platforms remains problematic due to (i) enrichment of ncRNA species that is below or exceed the detection limit (ii) amplification bias and (iii) false positive detection from non-full length RNA sequencing. For example, NanoString miRNA detection platform that utilize solution-based hybridization and fluorescent-based barcode digital counting system showed only moderate correlation [133], Spearman's p = 0.49, with NGS platform with Illumina TruSeq Small RNA protocol that underwent pre-amplification, followed by size-selection and multiplexed sequencing in each flow cells prior to sequencing.

Validation of results obtained by any of the aforementioned methods is necessary and this is usually done with qPCR. Although collection of larger sample numbers are achieved in multi-institutional studies, acquiring robust data is usually problematic in this setting due to technical differences in blood processing, RNA isolation and quantitation methods. Strict methodological standardization must be applied to generate informative circulating RNA data.

## 14 Clinical application of circulating RNA

Circulating cell-free RNA has a major potential as a cancer biomarker. A number of RNA species are deregulated as a result of the uncontrolled cell proliferation, stromal remodeling and immune regulation that define cancer. Distinct alteration in circulating RNA reflects dysregulation of cancer immunity, cell growth, proliferation and stromal interaction. Given the systemic nature of cancer, its biology should be studied in the context of the host response, which makes cf-RNAs suitable complementary tools. Besides the non-invasive nature of blood sampling, liquid biopsies allow for serial sample collection at different time points relative to treatments. This is particularly valuable with respect to the promising cancer immune therapy research that relies on the host response.

## 15 Summary and future direction of circulating biomarkers

Circulating biomarkers development is a fledgling but rapidly growing field in cancer research. Circulating biomarkers will continue to evolve with ongoing improvements in detection limits, decreasing the amount of nucleic acids template, expanding the number genes available for analysis and reduction of the operating cost and time. An overall estimation of tumor characteristic with a snapshot of circulating nucleic acids is no doubt going to support treatment decisions and monitoring of cancer due to the dynamic nature of the disease and its heterogeneity. However, the major challenge in biomarker discovery is its validation in prospective clinical studies to assess their impact. Finally, until each of them is thoroughly validated and compared with standardized assessment for treatment response (i.e. RECIST criteria) and overcome problems of standardization, tumor-liquid biopsy discrepancies and lead-time bias, circulating biomarkers are still experimental and represent an interesting set of research tools.

## Figures and Tables

**Table 1:**
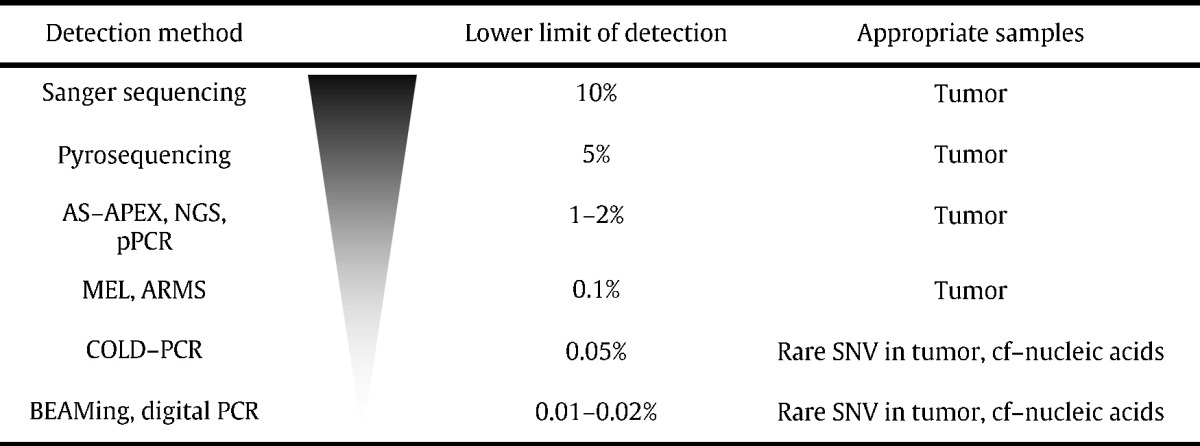
Limits of detection of nucleic acids by different methods. ARMS: amplification refractory mutation system; ASP-APEX: allele-specific arrayed primer extension; amplicon sequencing (review in [134]); BEAMing: bead, emulsion, amplification, magnetic polymerase chain reaction; cf: cell free; COLD-PCR: coamplification at lower denaturation temperature-PCR; ditigal PCR or ddPCR: droplet digital polymerase chain reaction; MEL: mutant enriched liquid chip; NGS: next-generation sequencing; Pyroseq: Sanger sequencing uses chain termination with dideoxynucleotide. Pyrosequencing relies on detection of pyrophosphate release during strand synthesis; qPCR: quantitative polymerase chain reaction; SNV: single nucleotide variant. Adapted from [135].

**Table 2:** Selected circulating cell-free DNA and RNA biomarkers in cancer.

DNA	Related cancer types	Treatment monitoring	Prognostic value	Predictive value
BRAF	CM	[47], [136]	[47], [136]	
PIK3CA	MBC	[137]		[138]
MGMT	CM, GBM		[139]	[44], [140]
KRAS	CRC, PDAC	[27], [141], [142]	[142], [143]	[142], [143]
TP53	TNBC, GCa	[144]	[145]	
ESR1	ER + BC, MBC	[146]	[147]	
EML4-ALK fusion	NSCLC			[148]
Personalized ctDNA	CRC, NSCLC	[31], [150]		


RNA markers	Related cancer types	Prognostic value	Predictive value
miR-125b-5p	MBC, DLBCL, NSCLC	[151], [152], [153], [154]	n/a
miR-155	CLL	[155]	n/a
miR-200	MBC, CRC, EOC	[156], [157], [158], [159], [160]	n/a
miR-21-5p	CRC, GCa, PDAC, MBC	[151], [161], [162], [163], [164], [165], [166], [167]	n/a
miR-210	CM, Pca, MBC	[168], [169], [170]	n/a
miR-221	CRC, PDAC, RCC	[171], [172], [173]	n/a
miR-222	GCa, GEC	[115], [174]	n/a

TNBC: triple-negative breast cancer, CM: cutaneous melanoma, GBM:glioblastoma multiforme, ER + BC: estrogen receptor positive breast cancer, CRC: colorectal cancers, PDAC: pancreatic ductal adenocarcinoma, MBC: metastatic breast cancer, NSCLC: non-small cell lung carcinoma, GCa: gastric cancer, GEC: gastro-esophageal cancer, PCa: prostate adenocarcinoma, RCC: renal cell carcinoma, EOC: epithelial ovarian carcinoma, DLBCL: diffuse large B-cell lymphoma, CLL: chronic lymphocytic leukemia.
